# Supplementary material for: Effective Carbon Dioxide Mitigation and Improvement of Compost Nutrients with the Use of Composts’ Biochar
Source: Materials (Basel). 2024 Jan 25;17(3):563. doi: 10.3390/ma17030563 (PMC10856095; doi:10.3390/ma17030563)
Supplement: Supplementary file 1 [file materials-17-00563-s001.zip › materials-2789630-supplementary.pdf]

Supplementary Material

1.1 Supplementary Figures

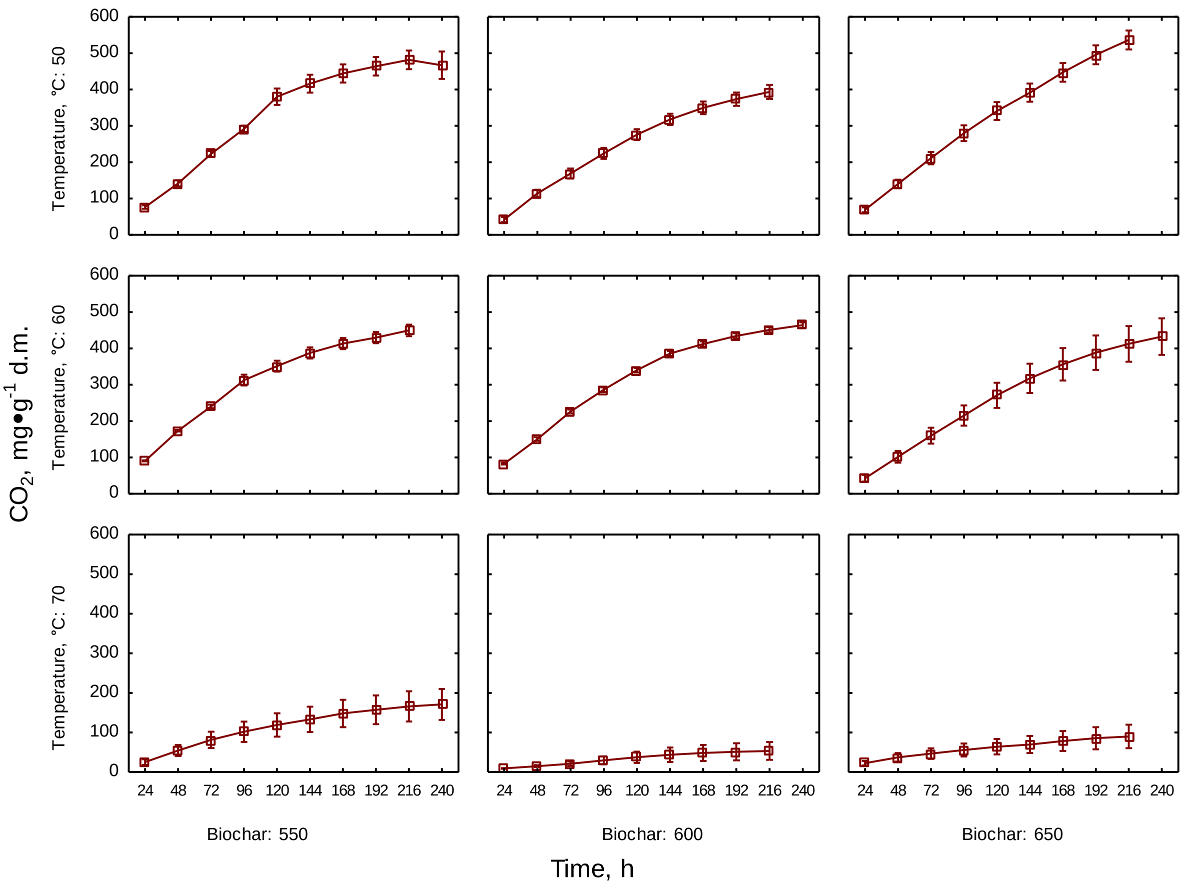

Supplementary Figure S1. Cumulative emission of CO_2 in 10 days of composting in different temperatures, and biochar type.

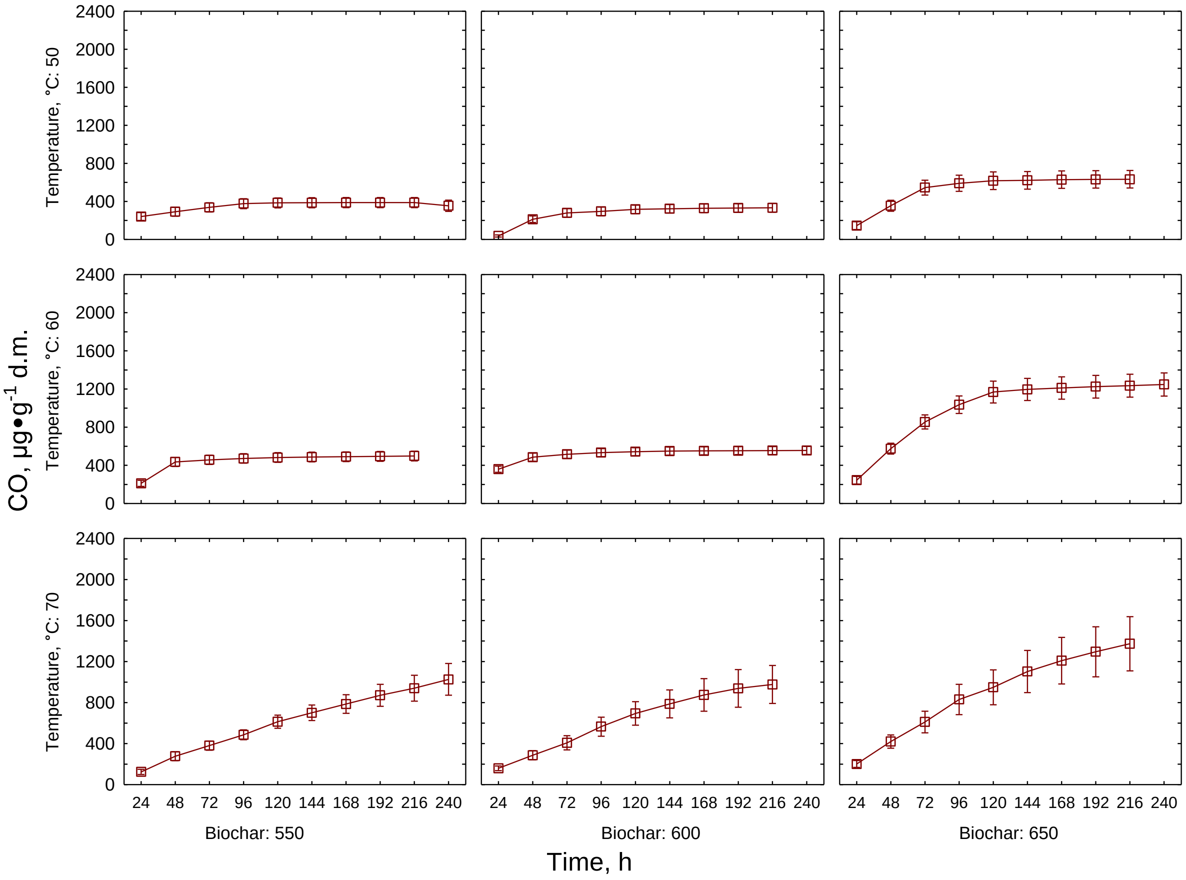

Supplementary Figure S2. Cumulative emission of CO in 10 days of composting in different temperatures, and biochar type.

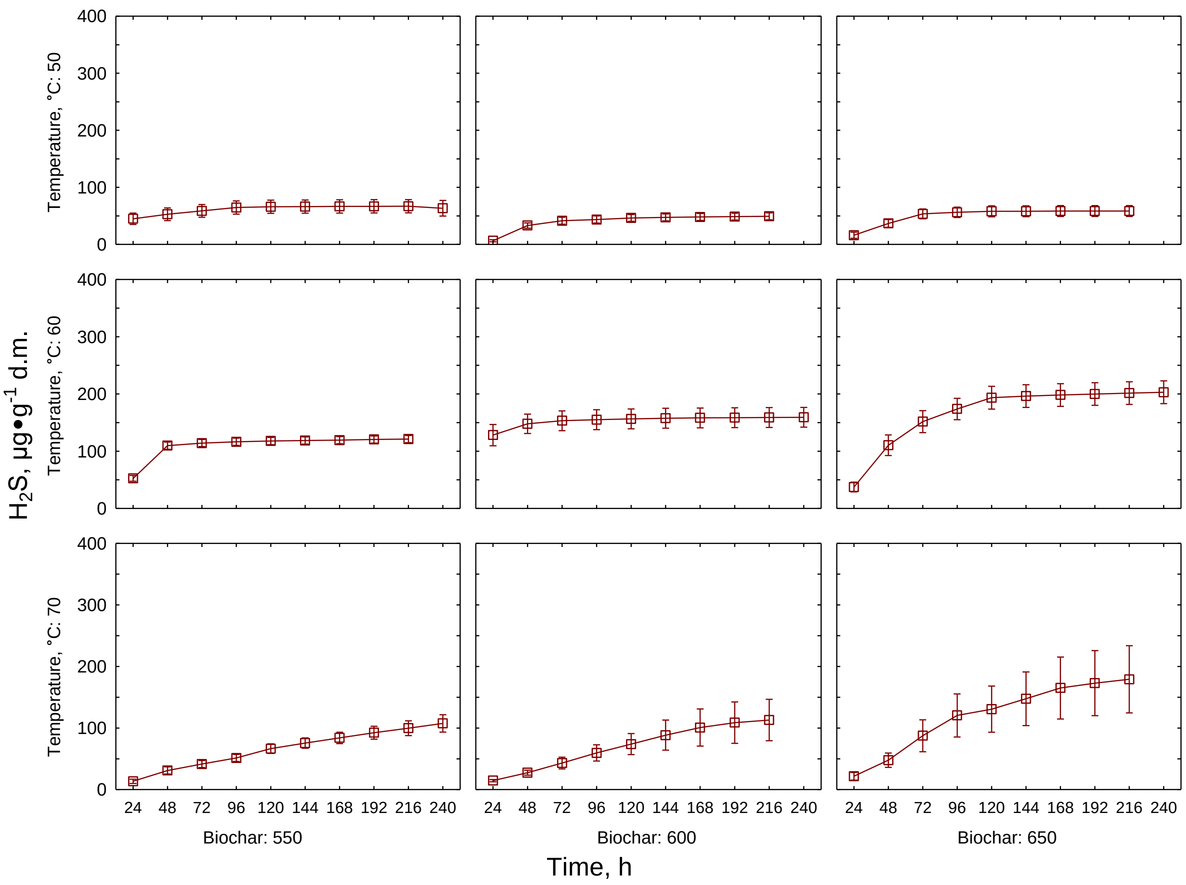

Supplementary Figure S3. Cumulative emission of H_2S in 10 days of composting in different incubation temperatures, and biochar type..

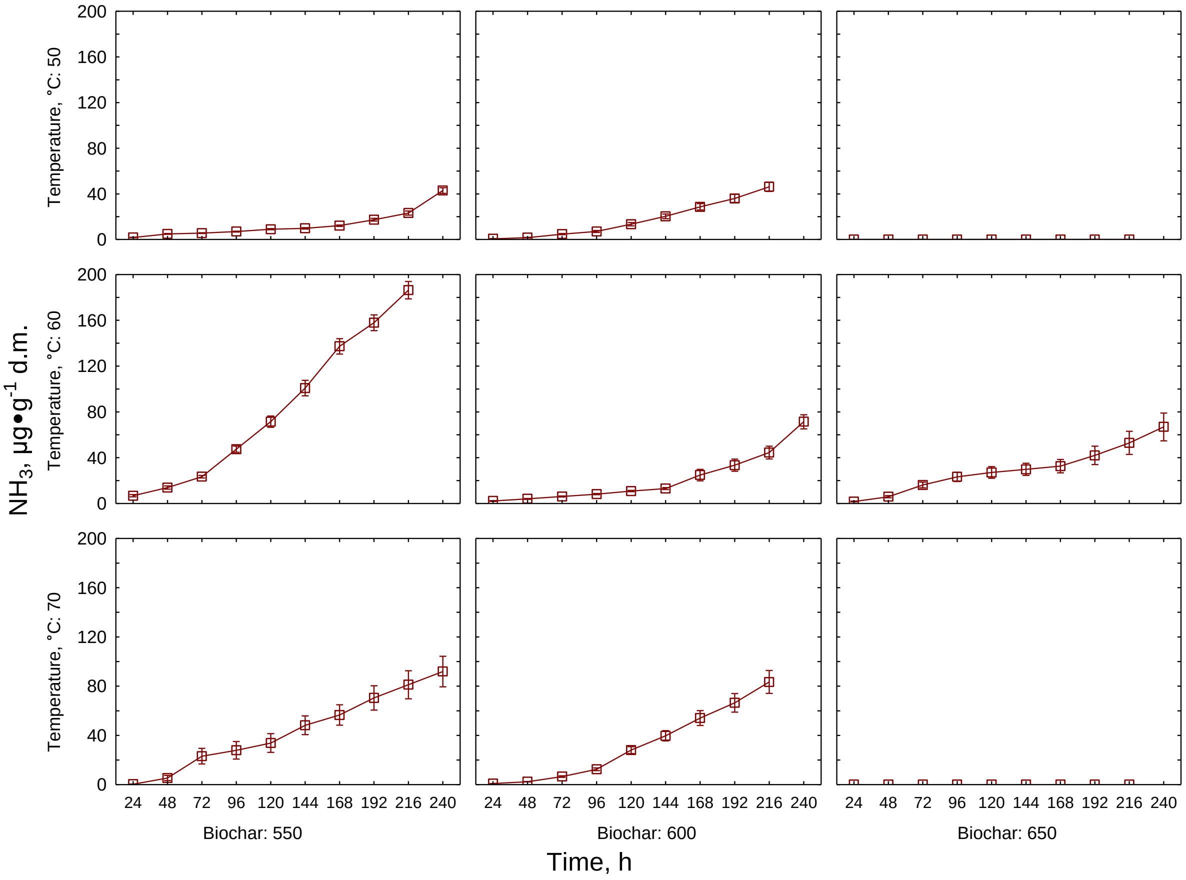

Supplementary Figure S4. Cumulative emission of NH_3 in 10 days of composting in different incubation temperatures, and biochar type.

A

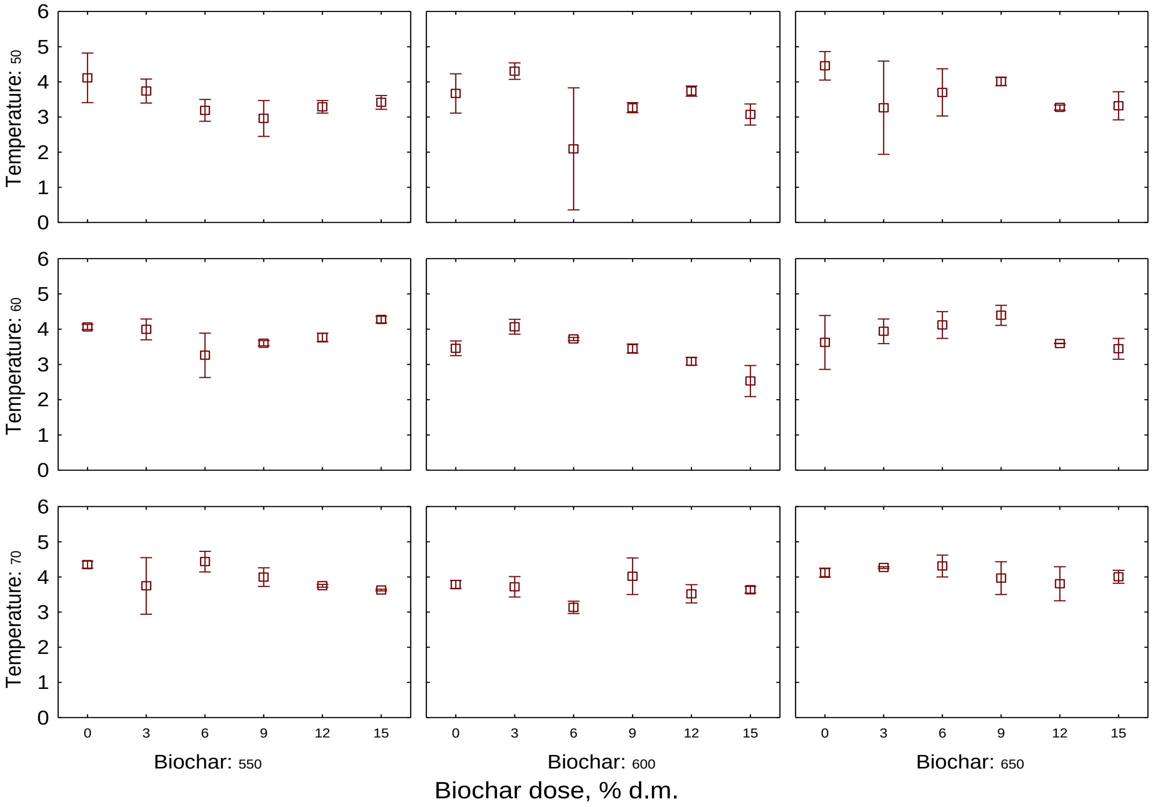
H, %
Temperature: 50
Temperature: 60
Temperature: 70
Biochar: 550
Biochar: 600
Biochar: 650
Biochar dose, % d.m.

B

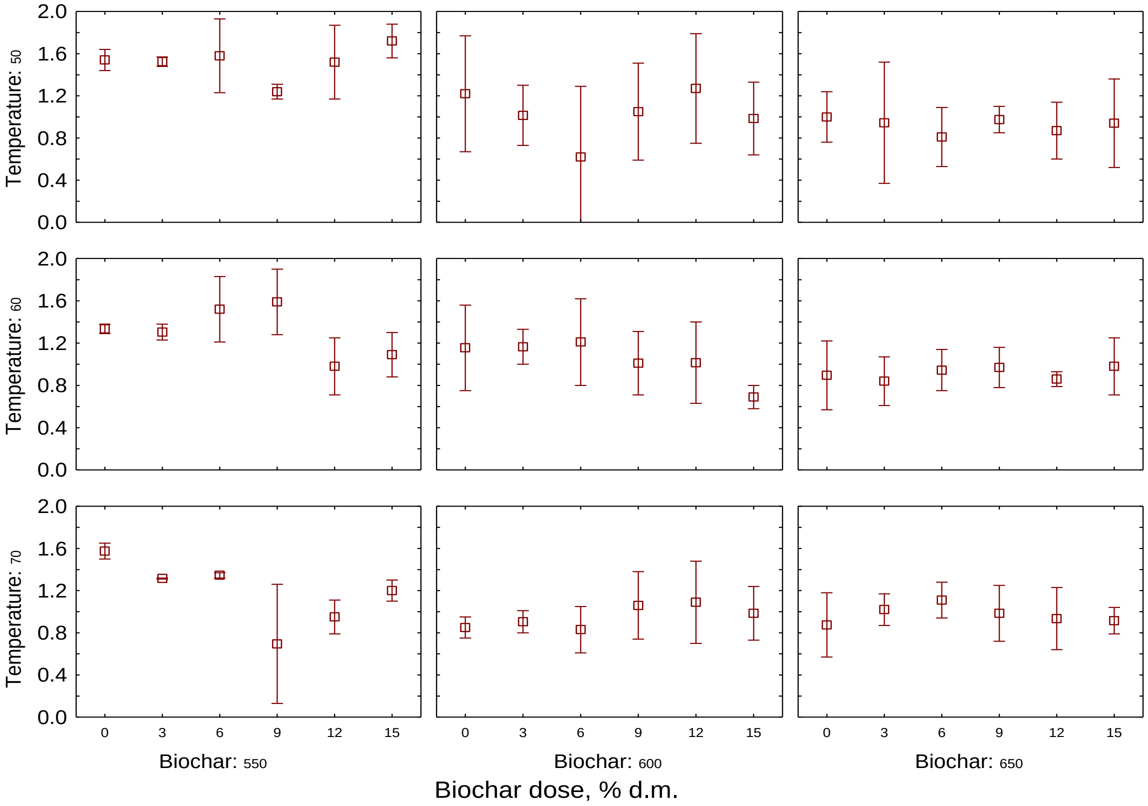
S, %
Temperature: 50
Temperature: 60
Temperature: 70
Biochar: 550
Biochar: 600
Biochar: 650
Biochar dose, % d.m.

Supplementary Figure S5.. Effect of biochar dose for (a) H, % and (b) S, %, during the first 10 days of composting process in laboratory scale; no statistical different were observed according to Tukey's test at significance level $p < 0.05$.

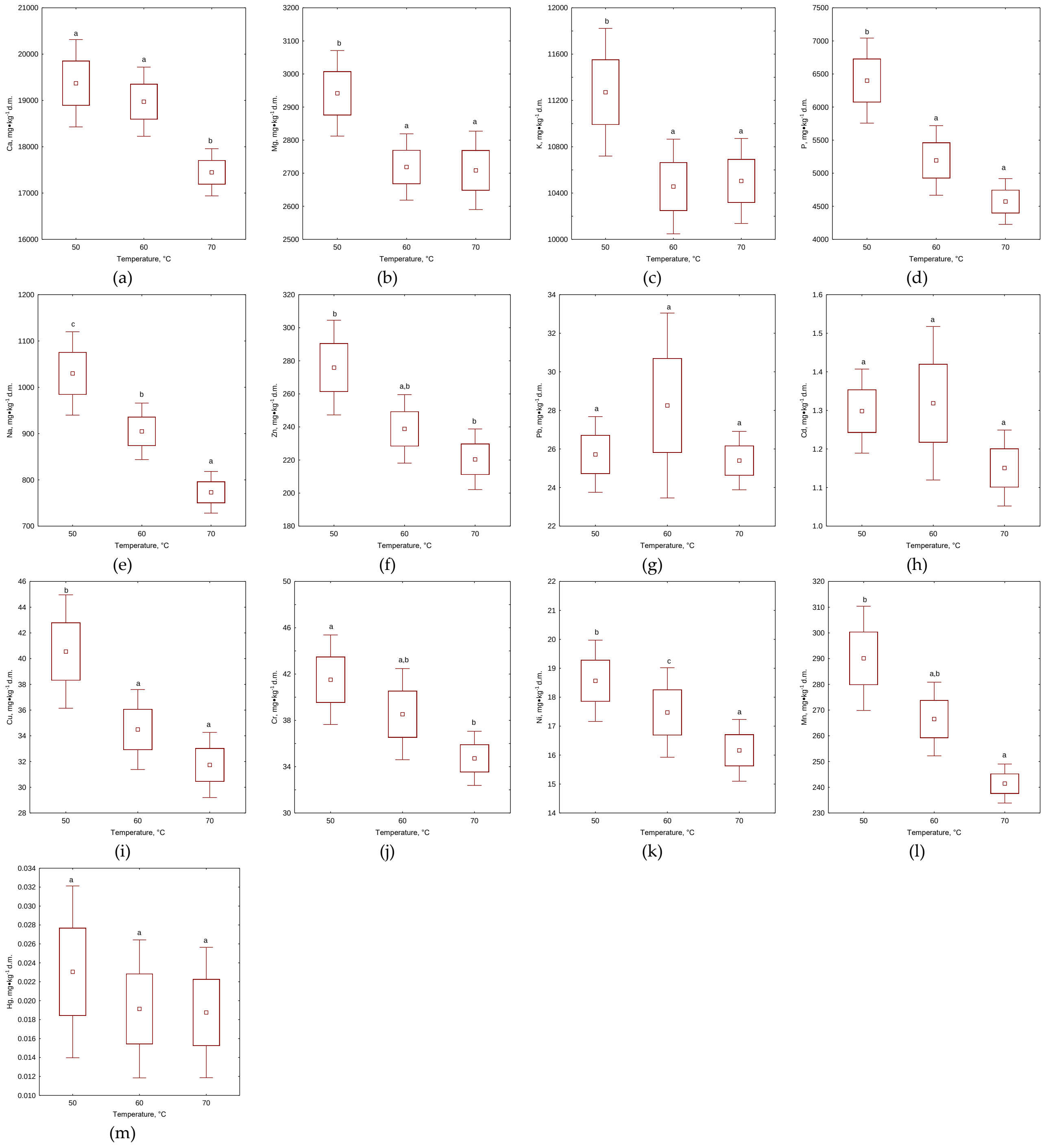

Supplementary Figure S6. Effect of incubation temperature for content of elements in substrate after 10 days of composting (a) calcium (Ca), (b) magnesium (Mg), (c) potassium (K), (d) phosphorus (P), (e) sodium (Na), (f) zinc (Zn), (g) lead (Pb), (h) cadmium (Cd), (i) copper (Cu), (j) chrome (Cr),(k) nickel (Ni), (l) manganese (Mn), (m) mercury (Hg)letters (a, b, c, d, e, f) indicate the homogeneity group according to Tukey's test at significance level $p < 0.05$.

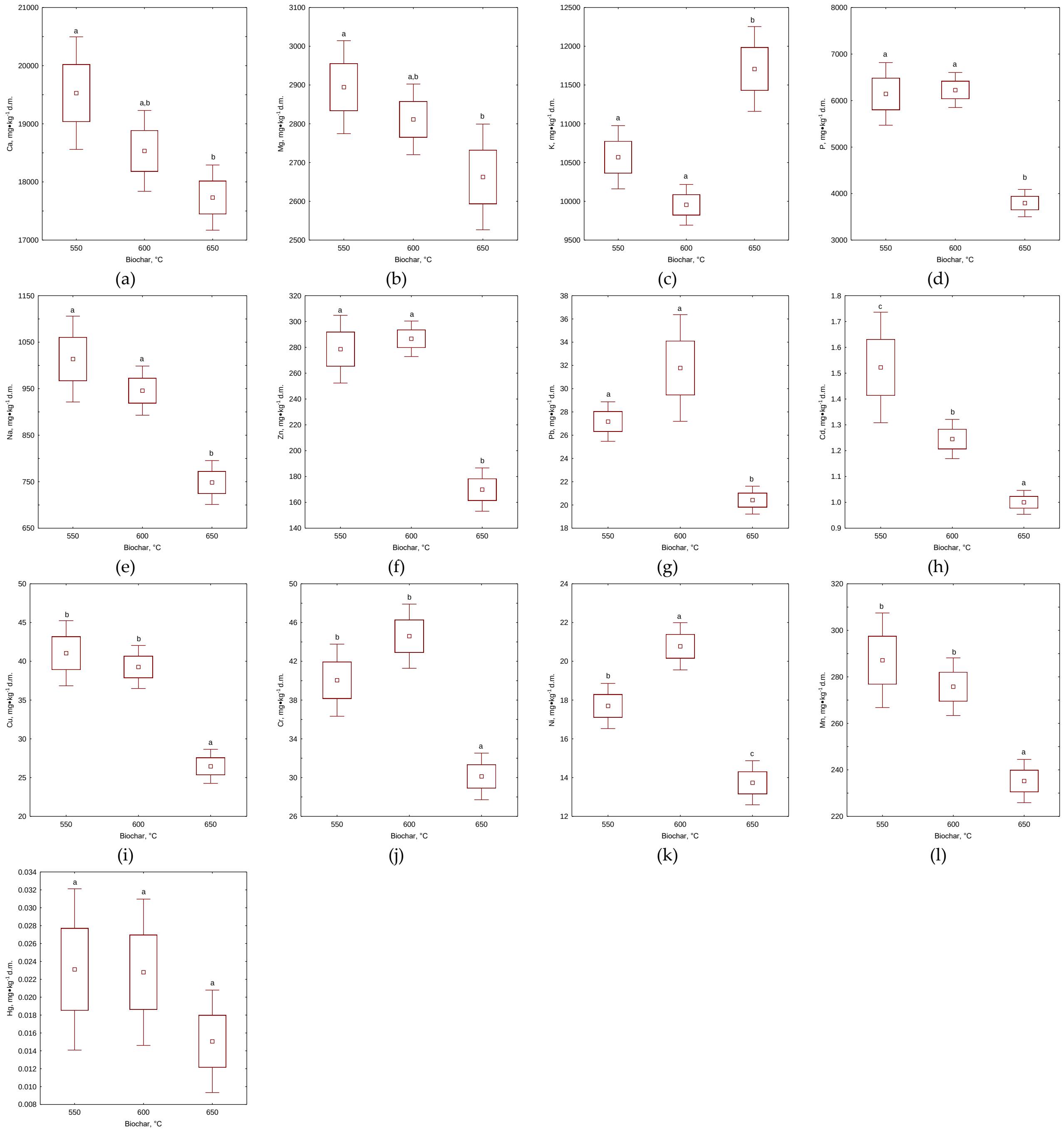

Supplementary Figure S7. Effect of biochar type (B550, B600, B650) for content of elements in substrate after 10 days of composting (a) calcium (Ca), (b) magnesium (Mg), (c) potassium (K), (d) phosphorus (P), (e) sodium (Na), (f) zinc (Zn), (g) lead (Pb), (h) cadmium (Cd), (i) copper (Cu), (j) chrome (Cr),(k) nickel (Ni), (l) manganese (Mn), (m) mercury (Hg), letters (a, b, c, d, e, f) indicate the homogeneity group according to Tukey's test at significance level $p < 0.05$.

Supplementary Tables

Supplementary Table S1. Total gas emissions after 10 days of the composting process in laboratory scale.

**Temperature**	**50 °C**																	
**Biochar**	**B550**						**B600**						**B650**					
**Biochar doses % d.m.**	**0**	**3**	**6**	**9**	**12**	**15**	**0**	**3**	**6**	**9**	**12**	**15**	**0**	**3**	**6**	**9**	**12**	**15**
**CO_2** mg·g^{-1} d.m.	482.9	406.7	458.4	521.1	436.5	584.7	429.1	380.4	412.2	355.7	415.0	368.9	565.0	568.3	532.4	473.6	471.0	643.3
**CO_2, % change**	-	-15.79	-5.07	7.9	-9.61	21.07	-	-11.35	-3.93	-17.1	-3.28	-14.02	-	0.6	-5.77	-16.17	-16.62	13.86
**CO** µg· g^{-1} d.m.	560.2	176.9	366.6	583.3	214.3	431.6	477.7	314.3	313.1	429.4	239.6	226.6	653.5	807.6	539.5	846.6	302.8	660.7
**CO, % change**	-	-68.42	-34.56	4.12	-61.76	-22.96	-	-34.19	-34.46	-10.11	-49.83	-52.56	-	23.58	-17.44	29.55	-53.67	1.1
**H_2S** µg· g^{-1} d.m.	85.5	28.0	50.7	116.9	57.7	63.5	61.6	50.4	44.1	57.0	36.5	45.9	60.4	83.3	42.8	73.1	25.3	70.7
**H_2S, % change**	-	-67.23	-40.73	36.64	-32.6	-25.76	-	-18.18	-28.38	-7.54	-40.71	-25.51	-	37.92	-29.05	21.13	-58.15	17.12
**NH_3** µg· g^{-1} d.m.	11.6	17.4	32.8	25.9	25.4	25.9	34.1	41.9	67.9	40.8	39.2	53.2	0.0	0.0	0.0	0.0	0.0	0.0
**NH_3, % change**	-	49.74	182.19	122.87	118.04	122.45	-	22.91	99.19	19.67	14.84	55.94	-	0	0	0	0	0
**Temperature**	**60 °C**																	
**Biochar**	**B550**						**B600**						**B650**					
**Biochar doses % d.m.**	**0**	**3**	**6**	**9**	**12**	**15**	**0**	**3**	**6**	**9**	**12**	**15**	**0**	**3**	**6**	**9**	**12**	**15**
**CO_2** mg· g^{-1} d.m.	410.8	433.2	492.2	435.0	467.2	459.2	484.8	480.4	446.6	460.7	491.4	409.0	522.5	542.8	617.9	377.0	322.6	212.2
**CO_2, % change**	-	5.45	19.83	5.91	13.73	11.78	-	-0.9	-7.87	-4.96	1.36	-15.64	-	3.89	18.25	-27.84	-38.27	-59.38
**CO** µg· g^{-1} d.m.	503.5	670.7	684.0	375.8	633.8	645.0	785.1	519.9	522.1	413.5	622.3	550.9	861.9	1120.7	1353.2	1491.8	1141.6	1515.7
**CO, % change**	-	33.2	35.85	-25.37	25.87	28.09	-	-33.78	-33.51	-47.34	-20.74	-29.83	-	30.03	57.01	73.09	32.46	75.87
**H_2S** µg· g^{-1} d.m.	129.4	133.9	152.1	76.8	116.5	118.8	191.5	103.9	144.0	150.2	235.2	133.0	171.8	249.9	215.6	170.7	193.7	216.9
**H_2S, % change**	-	3.46	17.54	-40.67	-10.02	-8.24	-	-45.74	-24.79	-21.6	22.8	-30.57	-	45.46	25.49	-0.64	12.76	26.27
**NH_3** µg· g^{-1} d.m.	188.5	205.7	199.1	215.1	150.8	159.1	59.0	70.5	106.5	72.5	59.4	49.4	159.2	99.3	58.5	35.2	25.9	23.4
**NH_3, % change**	-	9.15	5.62	14.15	-19.97	-15.58	-	19.48	80.58	22.84	0.69	-16.22	-	-37.62	-63.25	-77.88	-83.75	-85.27
**Temperature**	**70 °C**																	
**Biochar**	**B550**						**B600**						**B650**					
**% d.m.**	**0**	**3**	**6**	**9**	**12**	**15**	**0**	**3**	**6**	**9**	**12**	**15**	**0**	**3**	**6**	**9**	**12**	**15**
**CO_2** mg· g^{-1} d.m.	270.9	205.5	46.9	244.2	118.4	141.7	153.4	14.5	35.5	35.9	28.6	23.7	222.8	104.9	39.9	30.3	23.8	28.7
**CO_2, % change**	-	-24.14	-82.69	-9.88	-56.29	-47.71	-	-90.55	-76.85	-76.63	-81.36	-84.59	0	-52.9	-82.09	-86.42	-89.32	-87.13
**CO** µg· g^{-1} d.m.	687.4	1298.6	1845.5	875.6	667.0	796.5	784.1	360.7	780.5	1098.8	1693.3	980.6	508.8	424.1	1024.2	807.4	1963.4	2139.6
**CO, % change**	-	88.92	168.48	27.39	-2.97	15.87	-	-54	-0.46	40.13	115.95	25.06	-	-16.65	101.28	58.68	285.85	320.48
**H_2S** µg· g^{-1} d.m.	85.3	133.3	174.2	94.2	80.7	75.3	79.5	44.3	75.8	101.6	252.4	90.6	52.8	32.7	82.2	78.1	210.2	439.6
**H_2S, % change**	-	56.23	104.23	10.38	-5.41	-11.77	-	-44.33	-4.73	27.79	217.34	13.89	-	-38.03	55.66	47.85	298.01	732.46
**NH_3** µg· g^{-1} d.m.	43.1	61.9	97.9	67.3	124.7	173.6	119.1	114.0	94.6	67.8	52.0	45.4	0.0	0.0	0.0	0.0	0.0	0.0
**NH_3, % change**	-	43.67	127.15	56.23	189.53	302.95	-	-4.21	-20.52	-43.06	-56.36	-61.88	-	0	0	0	0	0

Supplementary Table S2. Characteristics of material after 10 days of incubation – pH and LOI.

Incubation temperature	Biochar type	Biochar dose, % d.m.	pH, -	LOI, % d.m.
50°C	B550	0	8.91	75.51
		3	9.1	76.29
		6	8.97	76.45
		9	8.93	66.58
		12	8.93	67.46
		15	9.01	76.37
	B600	0	8.36	75.95
		3	7.14	82.40
		6	7.94	79.63
		9	8.32	72.15
		12	8.97	74.48
		15	8.9	66.60
	B650	0	8.89	83.76
		3	8.70	81.24
		6	9.15	82.70
		9	8.99	77.36
		12	9.00	74.86
		15	9.01	76.30
60°C	B550	0	8.64	81.70
		3	8.83	81.38
		6	8.67	78.45
		9	7.76	80.56
		12	8.04	78.34
		15	8.75	81.82
	B600	0	8.22	77.85
		3	9.07	81.87
		6	8.63	79.35
		9	8.79	71.38
		12	8.93	70.92
		15	8.82	69.59
	B650	0	5.38	82.07
		3	5.1	74.49
		6	5.77	76.63
		9	5.51	73.53
		12	5.33	76.85
		15	8.54	75.82
70°C	B550	0	5.31	84.95
		3	5.32	77.78
		6	6.44	80.94
		9	5.87	76.66
		12	5.02	78.91
		15	8.59	71.79
	B600	0	6.3	77.85

		3	5.43	81.87
		6	7.28	79.35
		9	5.57	71.38
		12	5.94	70.92
		15	7.32	69.59
	B650	0	7.12	87.37
		3	7.84	81.68
		6	7.93	82.46
		9	8.68	81.10
		12	7.51	78.35
		15	7.61	81.91
